# A new efficacy parameter (complete/near complete symptom relief) in allergic rhinitis management: results with a new therapy MP29-02*

**DOI:** 10.1186/2045-7022-3-S2-P42

**Published:** 2013-07-16

**Authors:** Jean Bousquet, Glenis Scadding, David Price, Peter Hellings, Wytske Fokkens, Ullrich Munzel, Claus Bachert

**Affiliations:** 1 Hopital Arnaud de Villeneuve University Hospital, Montpellier, France; 2 The Royal National Throat, Nose and Ear Hospital, London, UK; 3 University of Abderdeen, Dept of General Practice & Primary Care, Aberdeen, UK; 4 University Hospitals Leuven, Dept of Otorhinolaryngology, Head and Neck Surgery, Leuven, Belgium; 5 Academic Medical Center, Dept of Otorhinolaryngology, Amsterdam, the Netherlands; 6 Meda Pharma, Biostatistics & Market Access, Bad Homburg, Germany; 7 Ghent University Hospital, Dept of Oto-rhinolaryngology, Ghent, Belgium

## Background

It is unclear what constitutes a clinically-meaningful response for allergic rhinitis (AR) outcomes. In a recent survey [1] most experts defined control as being "hardly troubled at all" by each symptom. We propose a new criterion of ≤1 point remaining in each nasal symptom score (Max AM+PM score for each symptom=6) of the reflective total nasal symptom score (rTNSS) to stringently test efficacy and provide an endpoint meaningful to physicians and patients. This criterion has been termed complete/near-to-complete symptom control. Any treatment providing this level of control (patients will feel "cured") should have considerable socioeconomic impact.

## Objective

To compare the proportion of patients achieving ≤1 point remaining in each of the 4 symptoms of the rTNSS (congestion, itching, rhinorrhoea & sneezing) and the time taken to achieve this response in patients treated with MP29-02* (a novel intranasal formulation of azelastine hydrochloride [AZE] and fluticasone propionate [FP]), FP, AZE or placebo (PLA) nasal sprays.

## Methods

610 patients (≥12 years old) with moderate-to-severe seasonal AR were randomized into a double-blind, placebo-controlled, 14 day parallel-group trial to receive MP29-02*, commercially-available AZE or FP nasal sprays or PLA nasal spray (all 1 spray/nostril bid; total daily dose [AZE: 548µg, FP: 200µg]). The primary outcome was change from baseline in rTNSS over 14-days. Time to achieve ≤1 point remaining in each nasal symptom (AM + PM) of the rTNSS was assessed post-hoc by Kaplan-Meier estimates and log rank tests.

## Results

17.8% of MP29-02* patients (1 out of 6) achieved this response versus 8.3%, 9.2% and 7.8% of those treated with AZE, FP and PLA, respectively. MP29-02* patients achieved this response up to 7 days faster than AZE (p=0.0152) and up to 8 days faster than either FP (p=0.0262) or PLA (p=0.0094). Neither AZE nor FP differed from PLA for this parameter.

## Conclusion

MP29-02* provides faster and more complete symptom control than first-line therapies for AR. One out of 6 moderate to severe AR patients achieved complete/near-to-complete and uniform symptom relief days faster than either FP or AZE. MP29-02* is the drug of choice for AR treatment since it was the only therapy to rapidly provide such a level of symptom control. This endpoint should become a new standard in assessing the efficacy of current and novel AR therapy.

*Dymista
